# Supplementary material for: Induced Proteinuria Enhances Adeno-Associated Virus Transduction of Renal Tubule Epithelial Cells After Intravenous Administration
Source: bioRxiv. 2025 Jun 2:2025.05.28.656514. Preprint. [Version 2] doi: 10.1101/2025.05.28.656514 (PMC12154760; doi:10.1101/2025.05.28.656514)

Supplemental Fig. 1. Rubin et al.

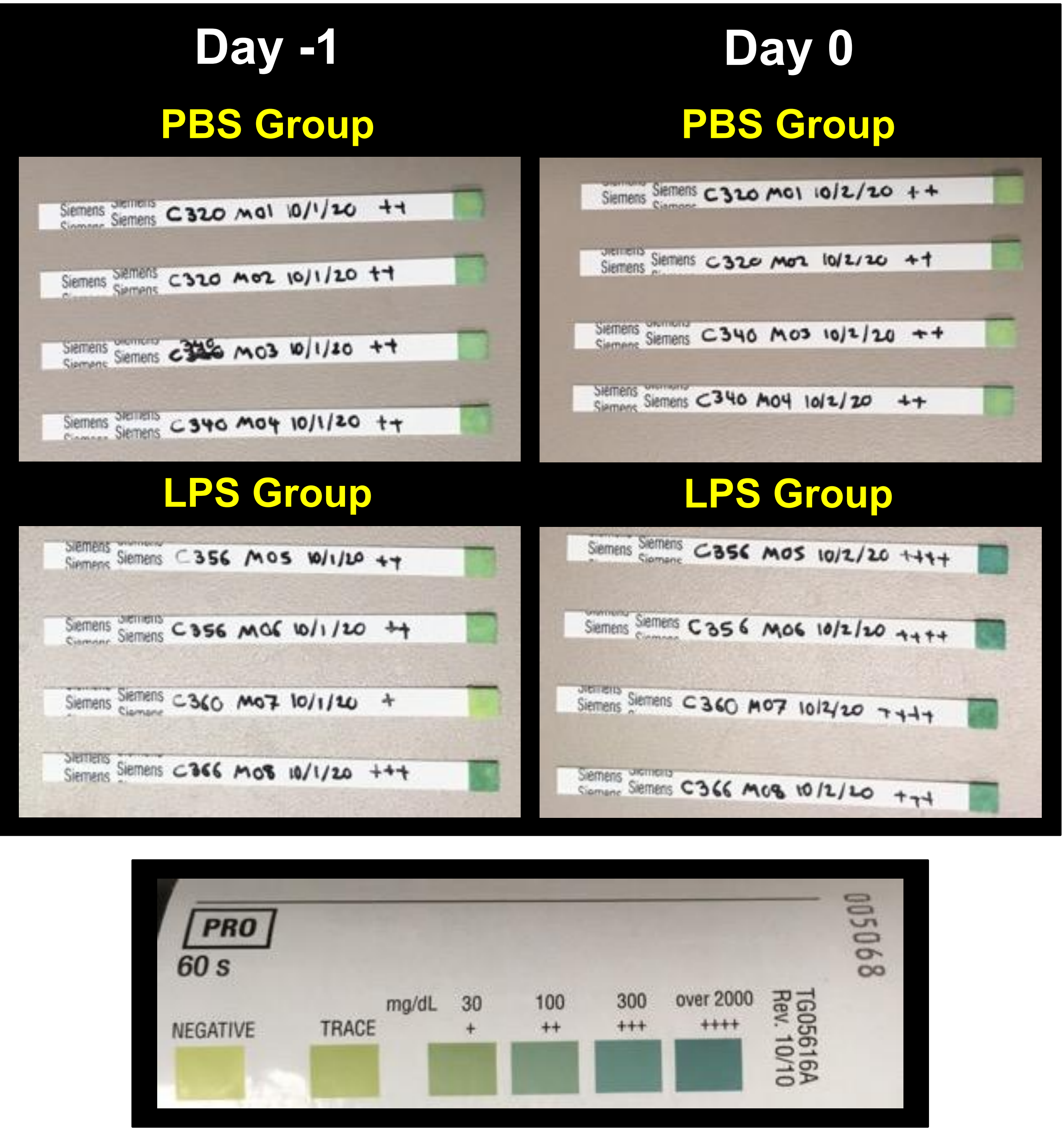

Supplemental Fig. 2. Rubin et al.

A

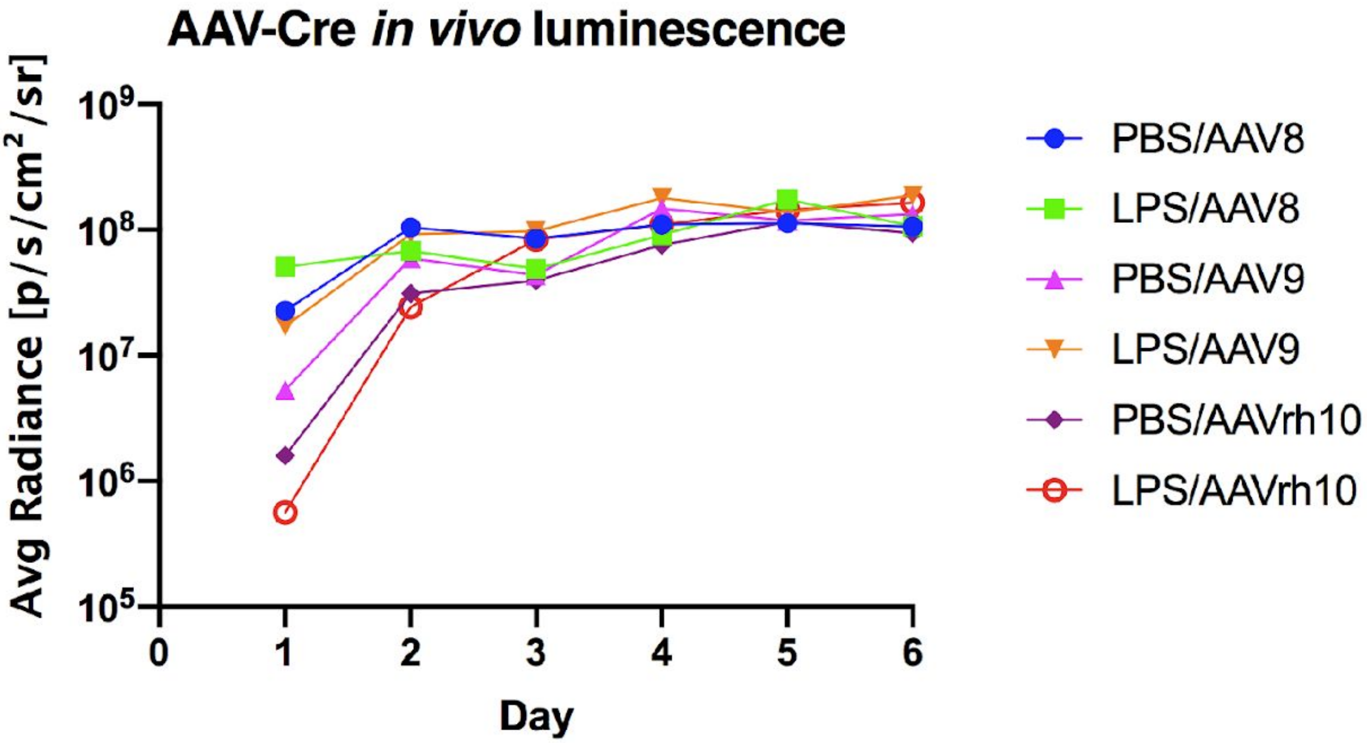

B

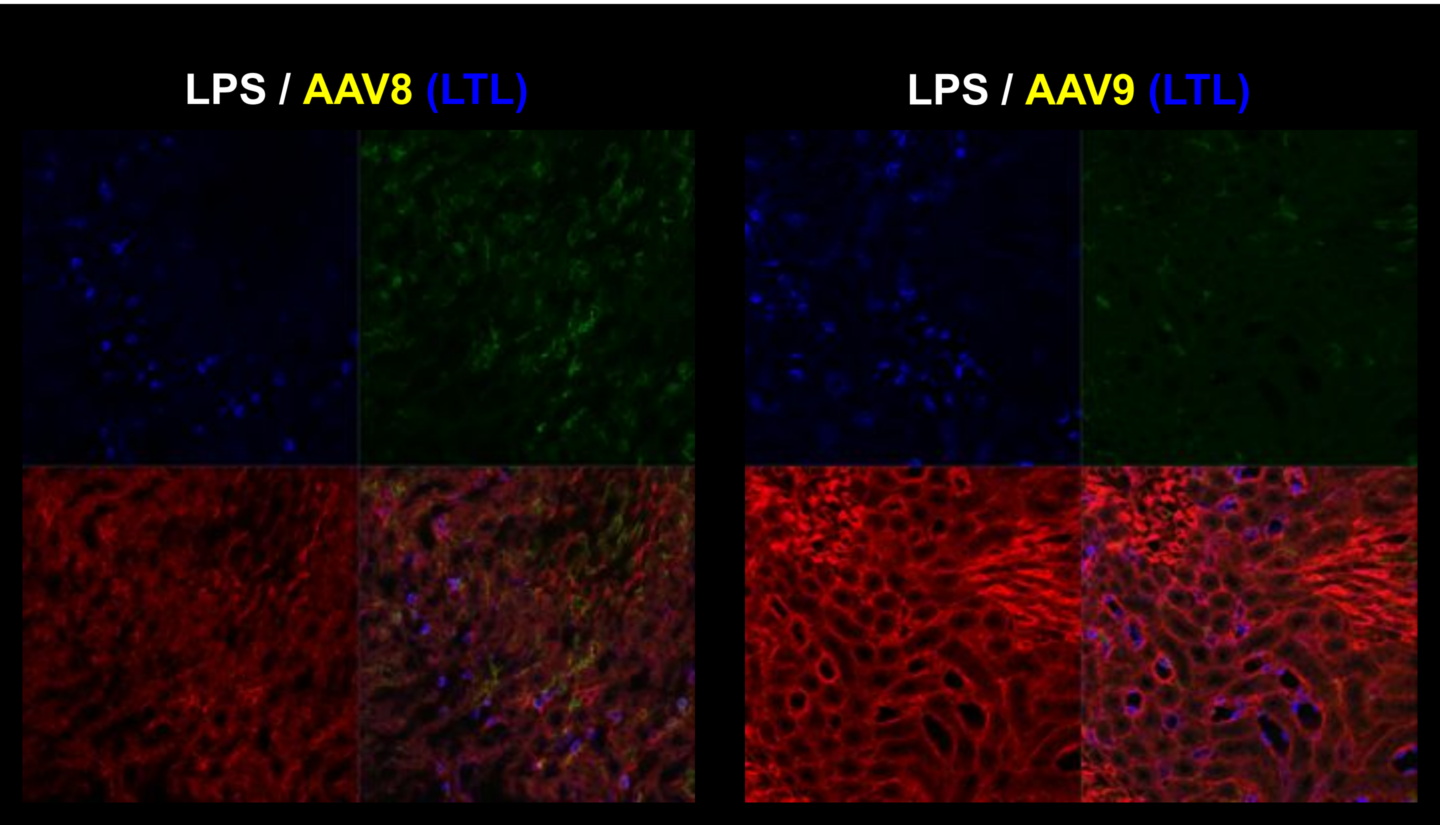

Supplemental Fig. 3. Rubin et al.

A

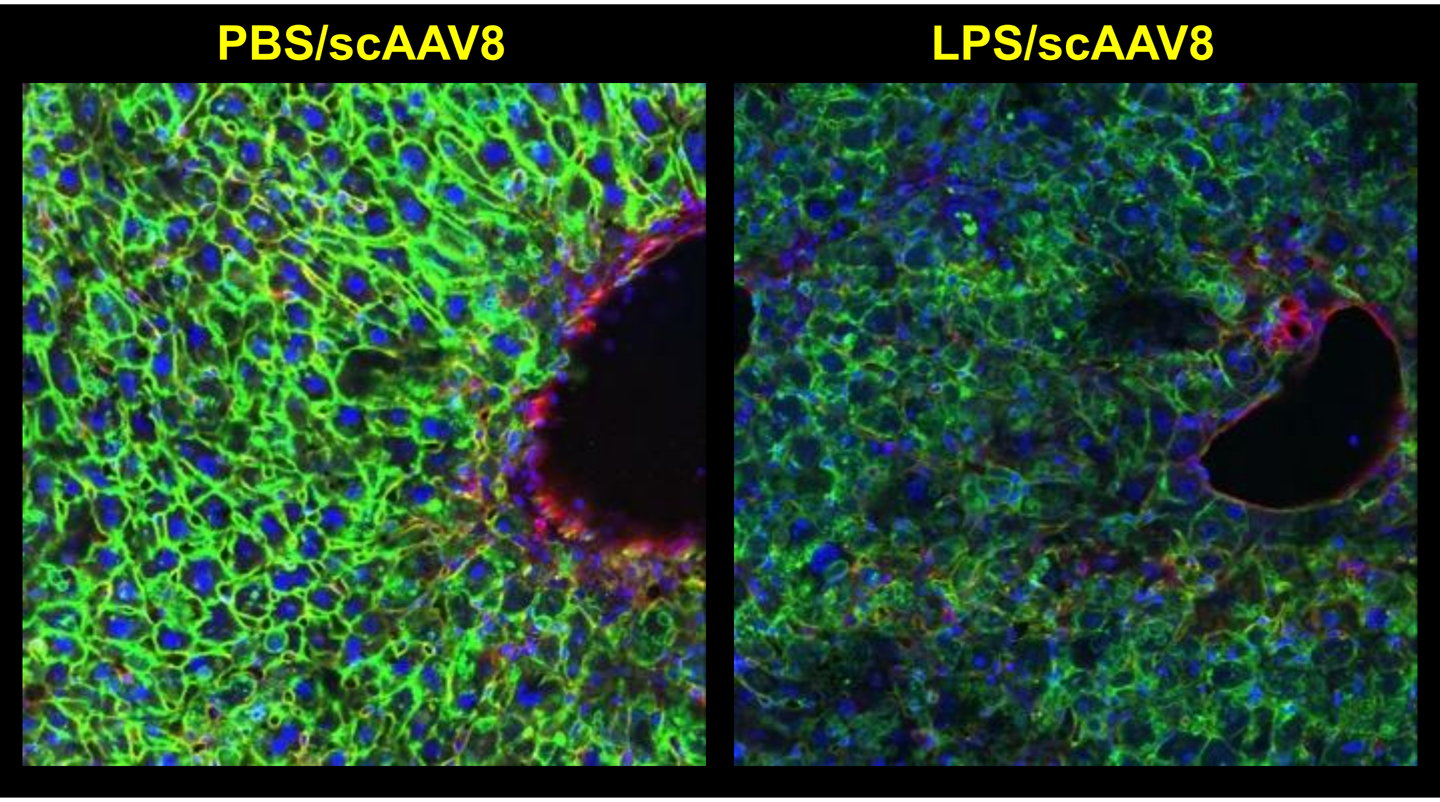

B

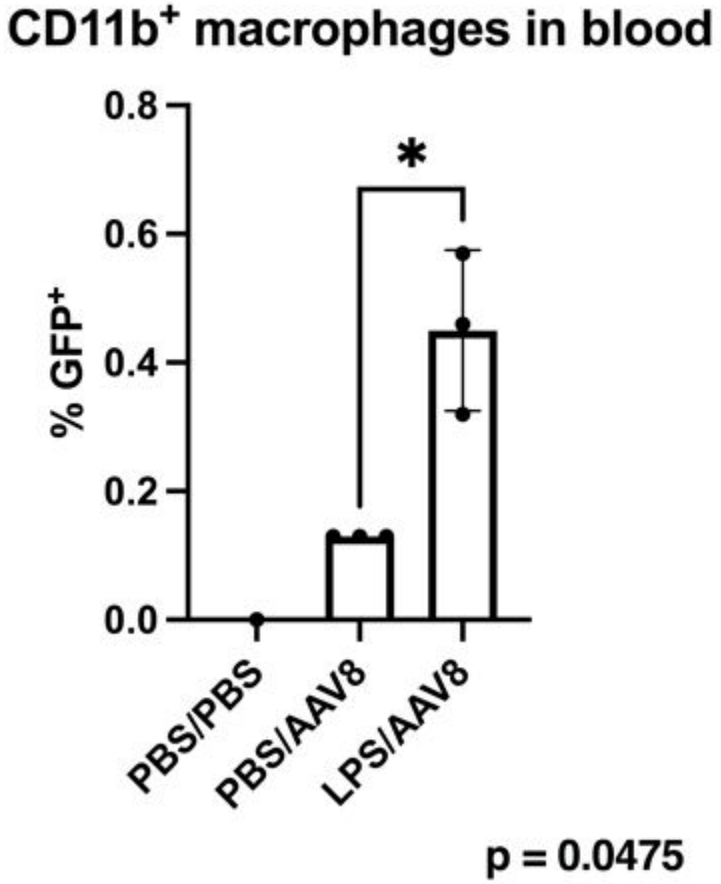

Supplemental Fig. 4. Rubin et al.

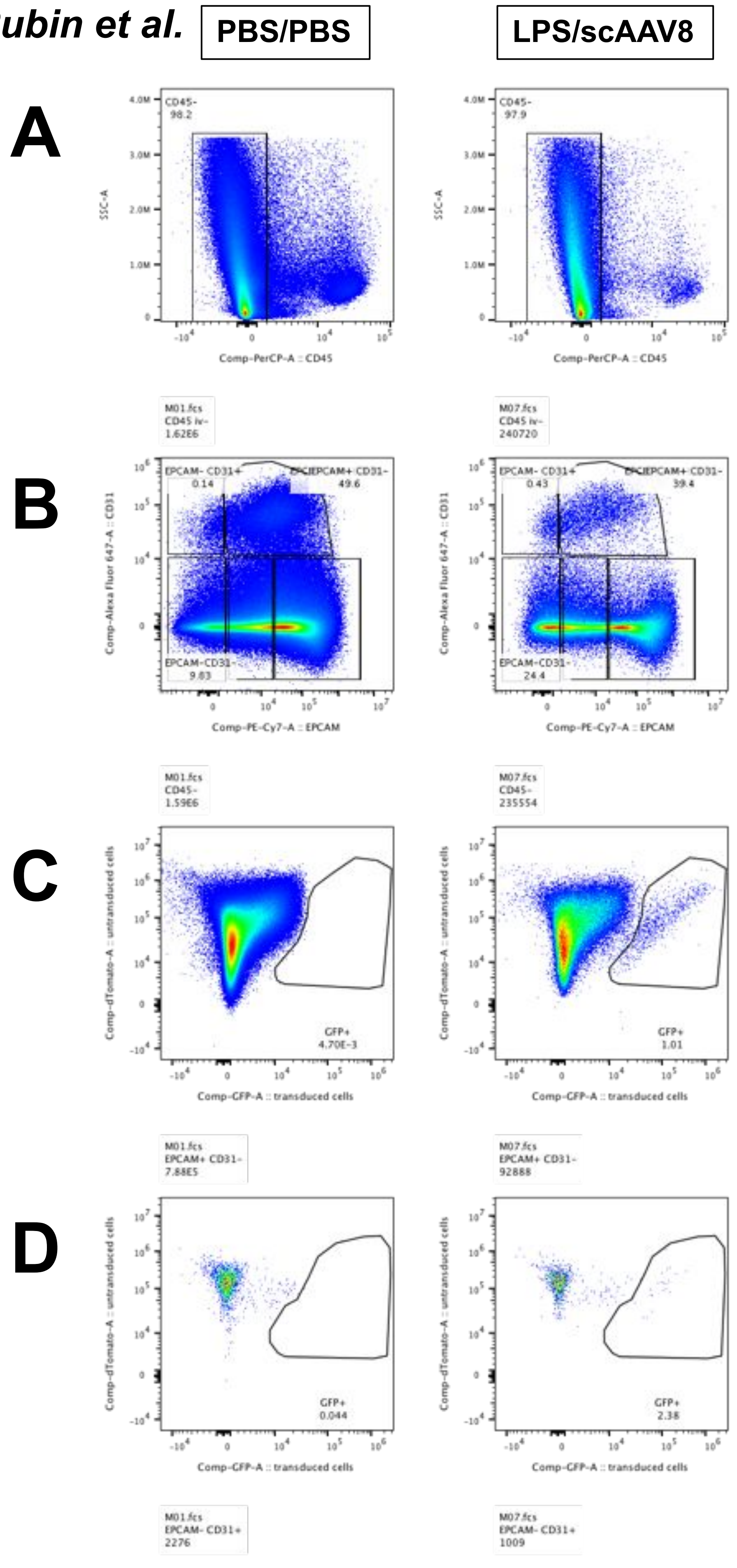

Supplemental Fig. 5. Rubin et al.

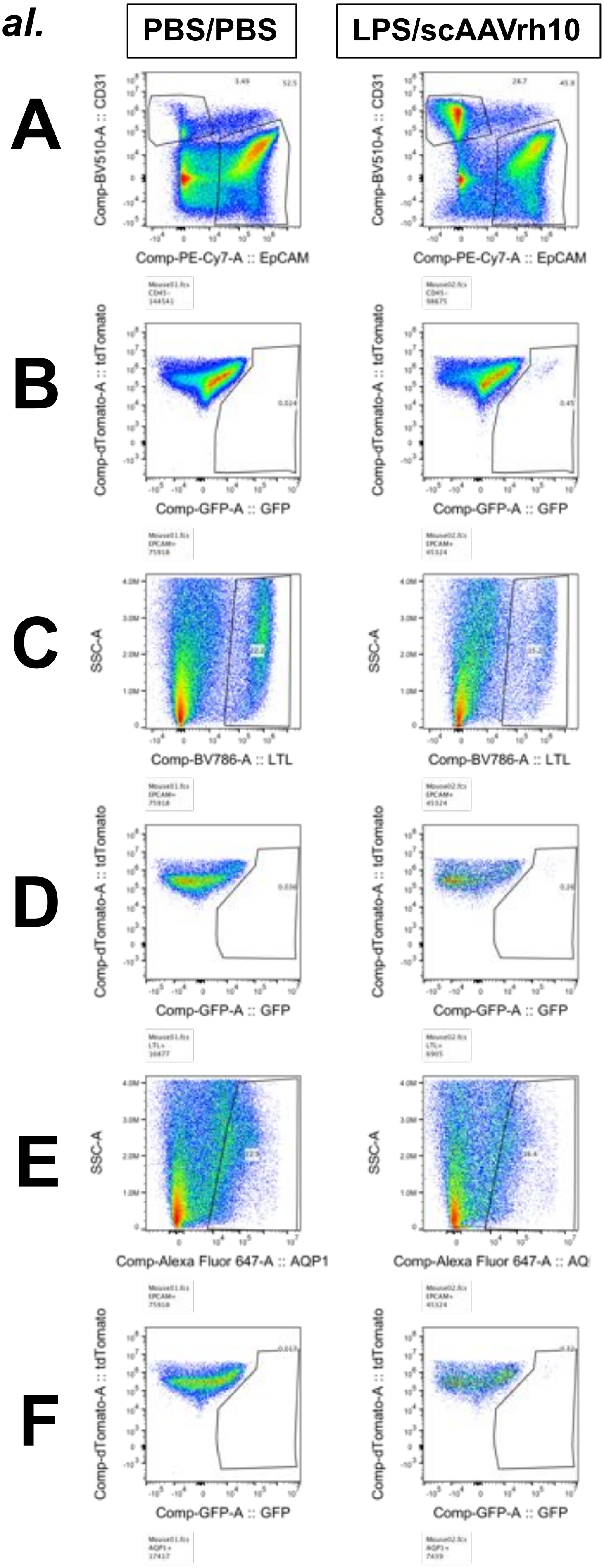

Supplemental Fig. 6. Rubin et al.

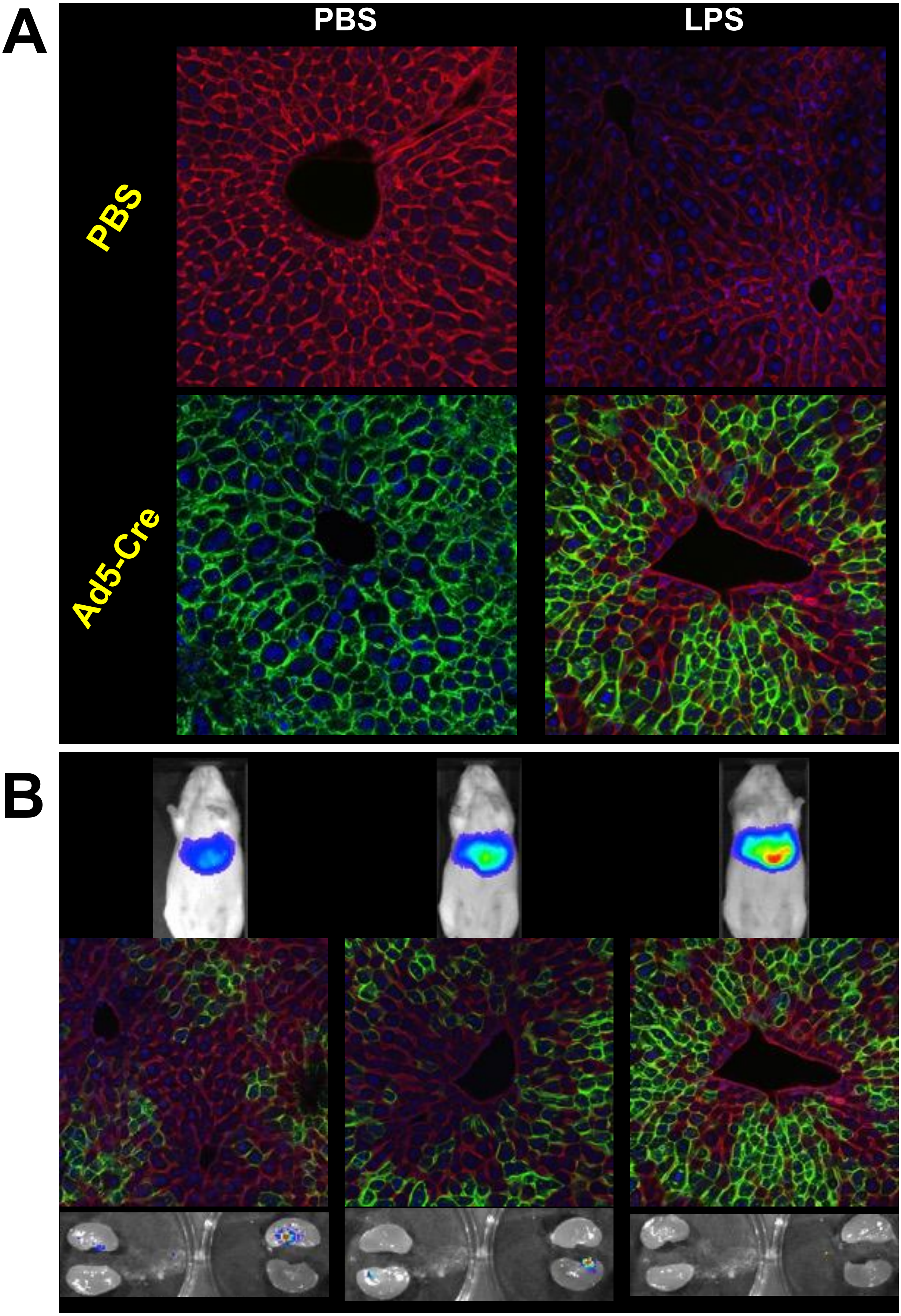

Supplemental Fig. 7. Rubin et al.

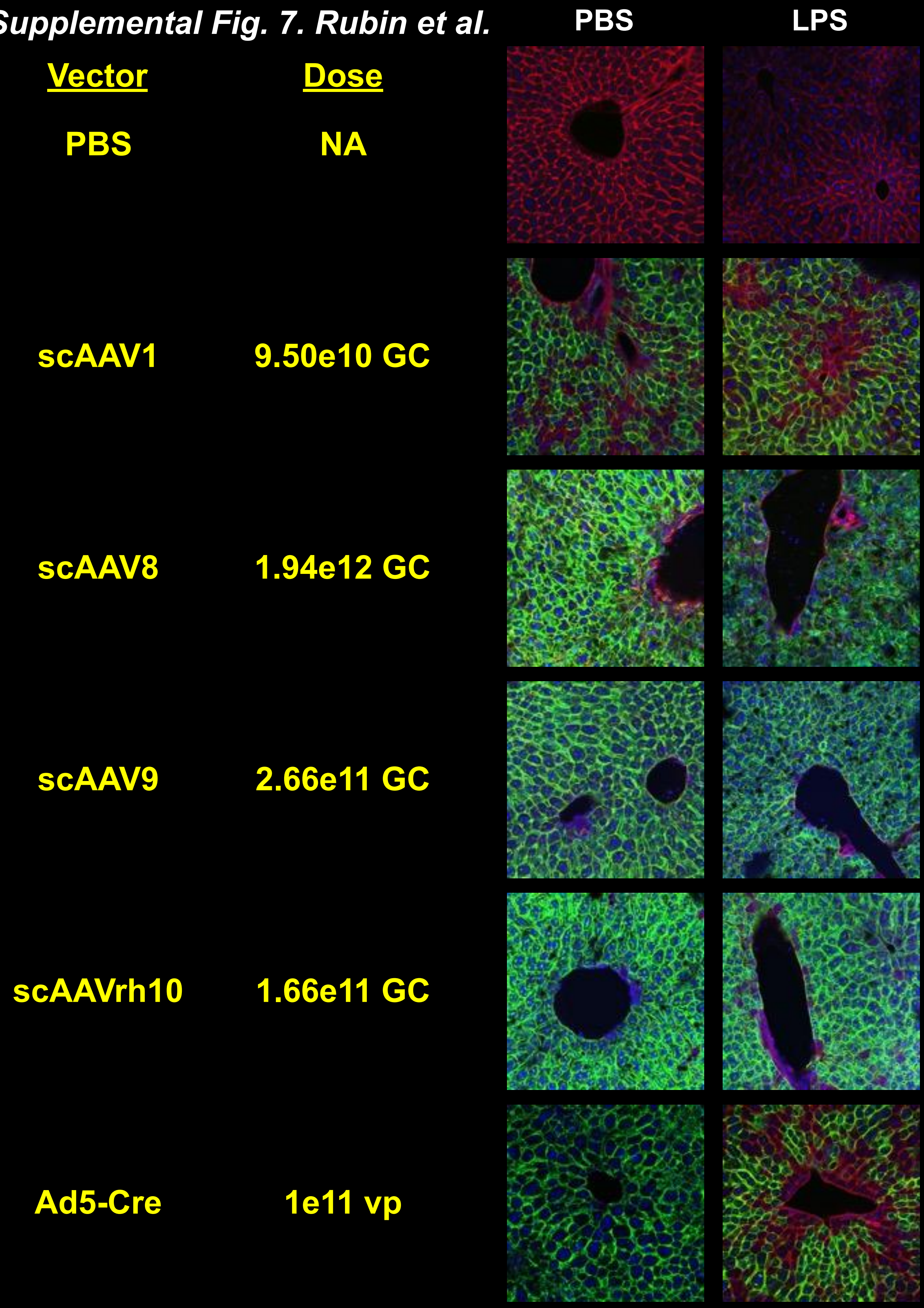

Supplement: 1 — Supplemental Fig. 1 Example of proteinuria dipsticks used to assess induced proteinuria in mice. Supplemental Fig. 2 Administration of LPS to mice did not affect liver transduction by AAV but did result in renal medullar transduction across several serotypes of AAV. Supplemental Fig. 3 Evidence of toxicity associated with combined LPS and AAV administration. Supplemental Fig. 4 Representative flow cytometry plots for mice administered scAAV8-Cre. Supplemental Fig. 5 Representative flow cytometry plots for mice administered scAAVrh10-Cre. Supplemental Fig. 6 Increased kidney transduction after administration of LPS and Ad5-Cre is negatively correlated with liver transduction. Supplemental Fig. 7 Comparison of liver transduction across various vectors and doses. [file NIHPP2025.05.28.656514V2-supplement-1.pdf]
